# Supplementary material for: Exploring Structural Diversity among Adhesion Devices Encoded by Lactococcal P335 Phages with AlphaFold2
Source: Microorganisms. 2022 Nov 16;10(11):2278. doi: 10.3390/microorganisms10112278 (PMC9692632; doi:10.3390/microorganisms10112278)

Supplementary Figure S1. Plots of the pLDDT values (y-axis) *vs* the protein sequence (x-axis). (A) Structure predictions of proteins from P335 phages types I and II. **(B)** Structure predictions of proteins from P335 phages types III and IV. The red dots squares identify the Dit arm-hand domain interacting with the RBP N-terminal domain. **(C)** Structure predictions of the NPS proteins from phages PhiLC3, TP901-1 and Tuc2009.

A

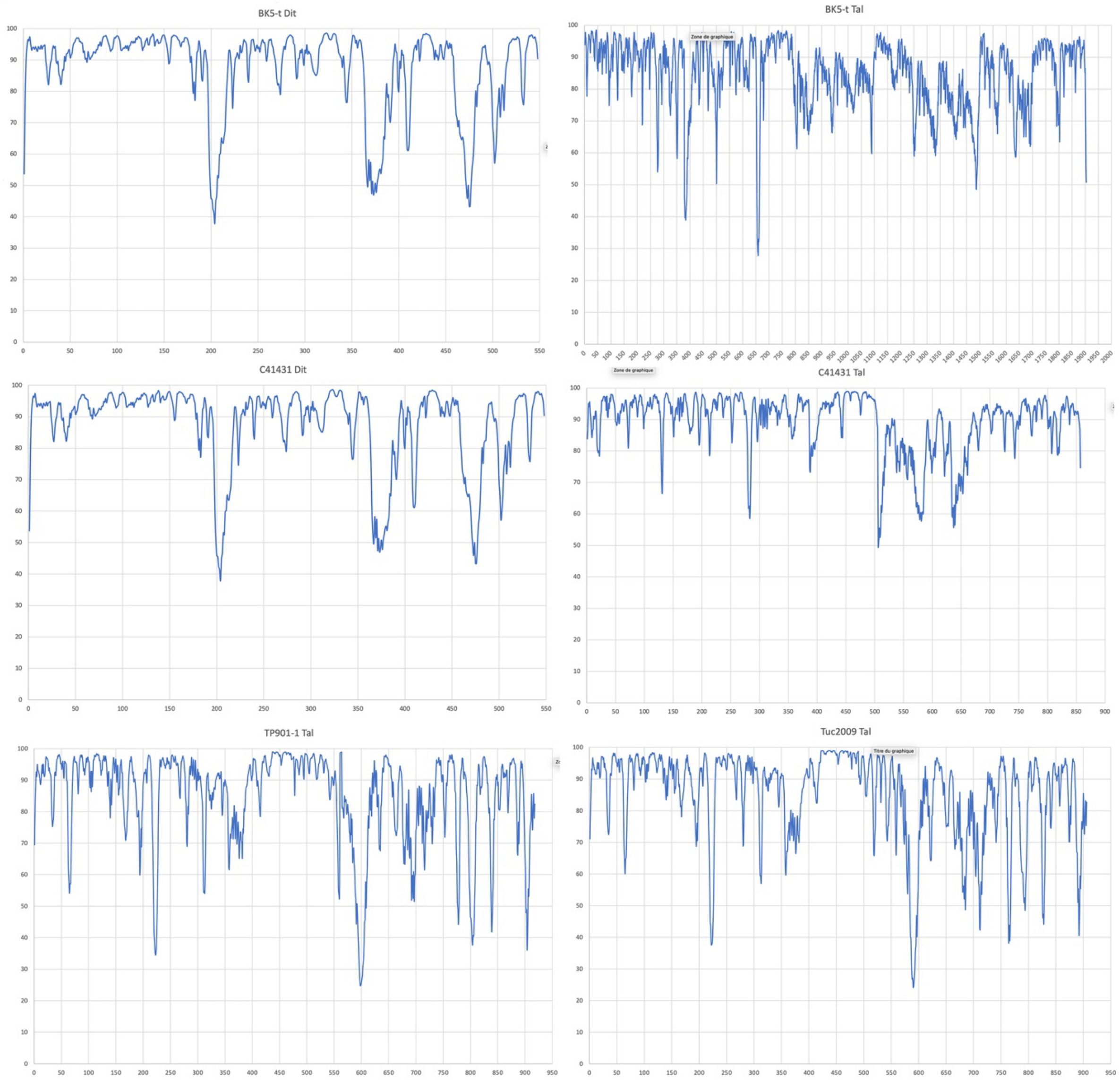

B

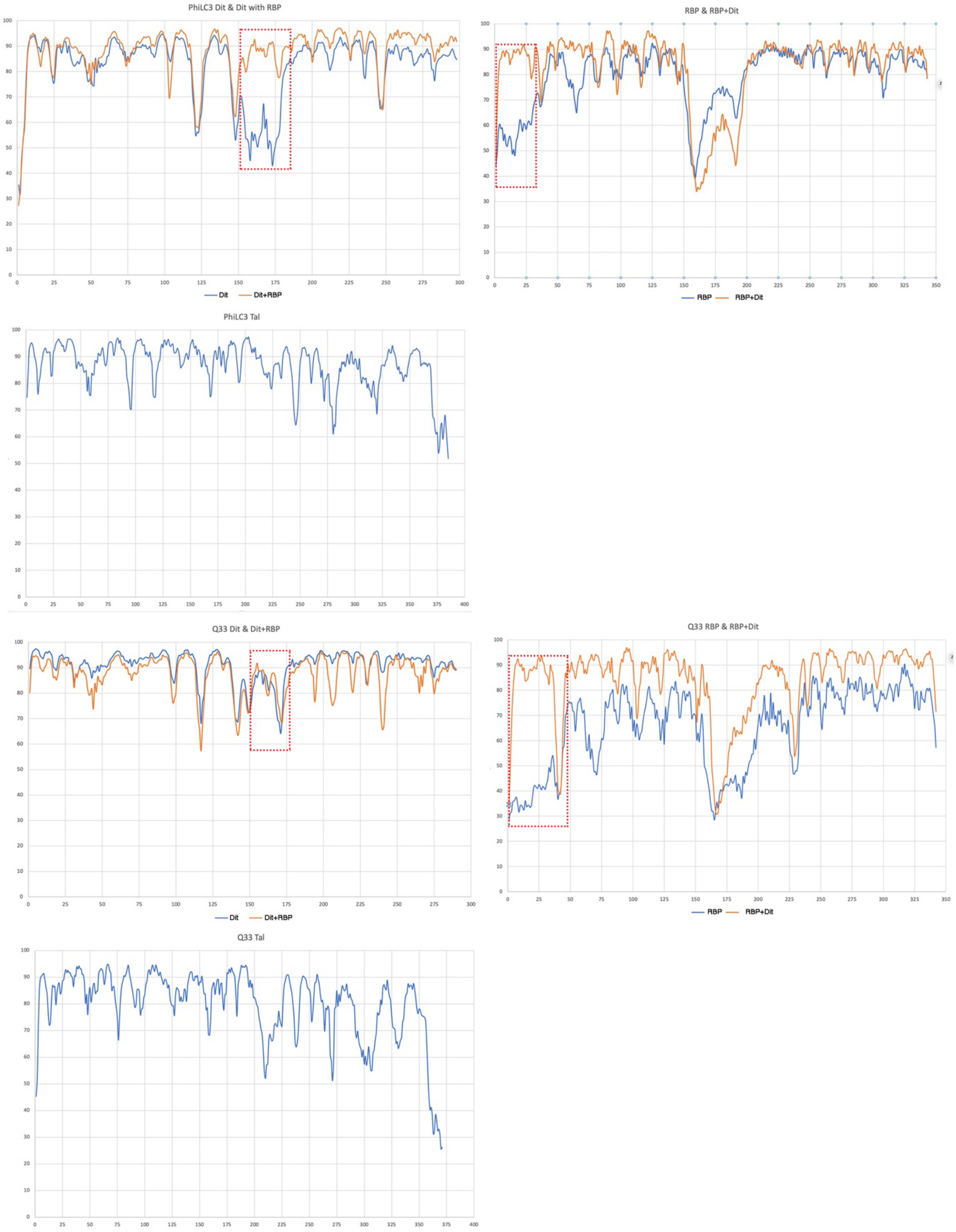
PhiLC3 Dit & Dit with RBP
Dit
Dit+RBP
RBP & RBP+Dit
RBP
RBP+Dit
PhiLC3 Tal
Q33 Dit & Dit+RBP
Dit
Dit+RBP
Q33 RBP & RBP+Dit
RBP
RBP+Dit
Q33 Tal

C

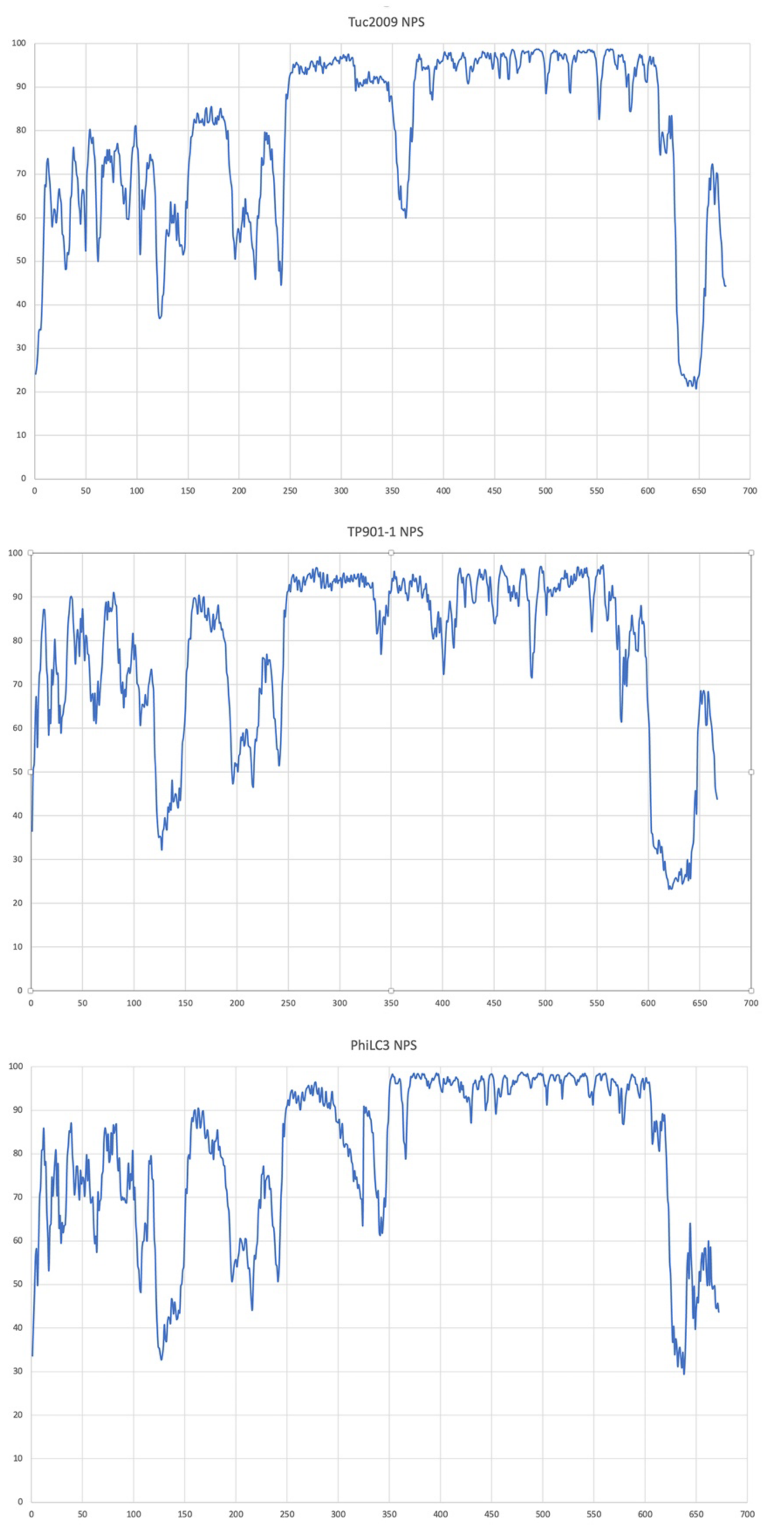
Tuc2009 NPS
100
90
80
70
60
50
40
30
20
10
0
0 50 100 150 200 250 300 350 400 450 500 550 600 650 700
TP901-1 NPS
100
90
80
70
60
50
40
30
20
10
0
0 50 100 150 200 250 300 350 400 450 500 550 600 650 700
PhiLC3 NPS
100
90
80
70
60
50
40
30
20
10
0
0 50 100 150 200 250 300 350 400 450 500 550 600 650 700

Supplementary Figure S2. Sequence alignment of adhesion device proteins from phages TP901-1 and Tuc2009

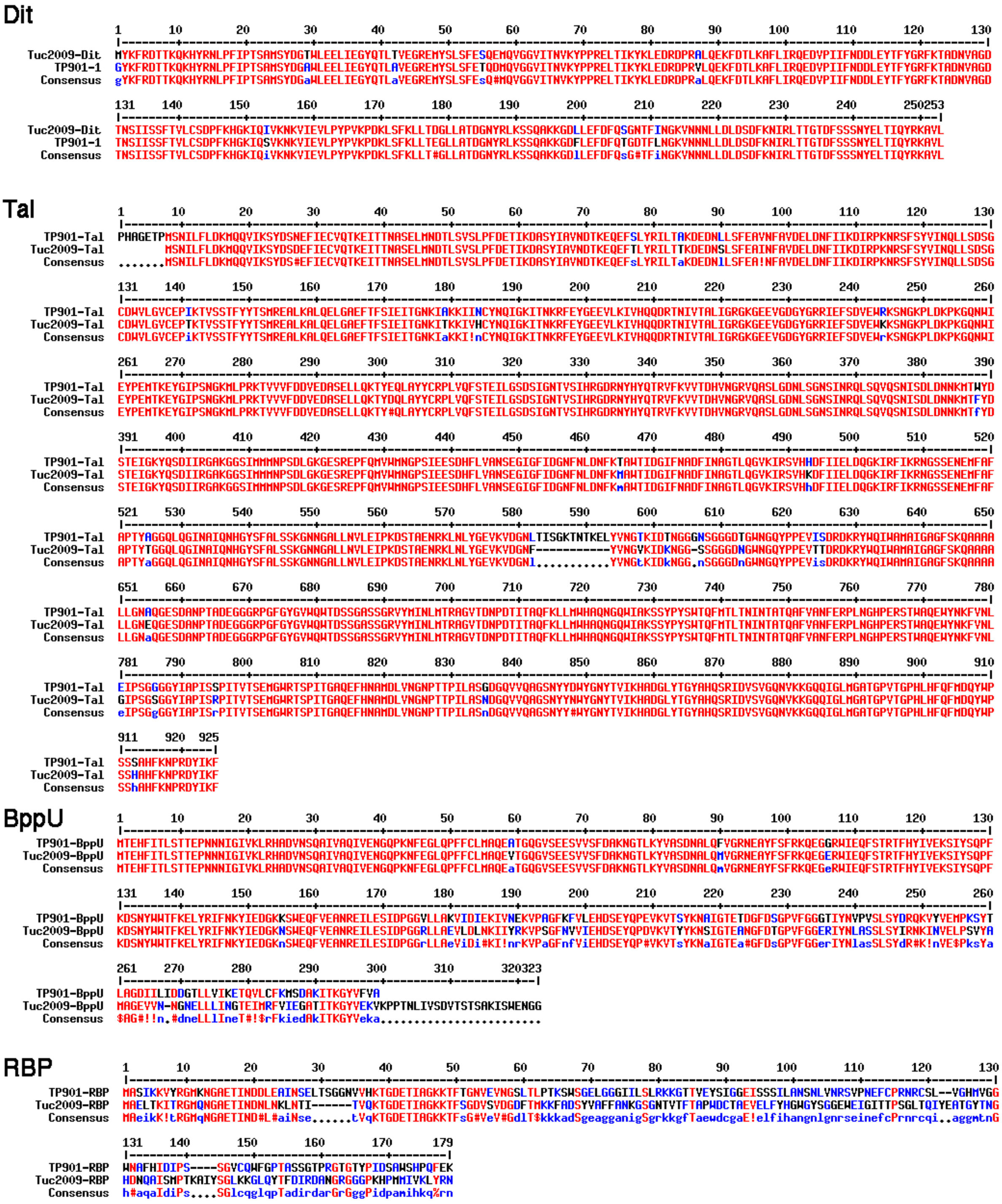

Supplementary Figure S3. **Sequence alignment of RBP sequences**. **(A)** phage TP901-1 *vs* PhiLC3. Residues involved in receptor binding are identified by red dots. **(B)** Tuc2009 *vs* Q33.

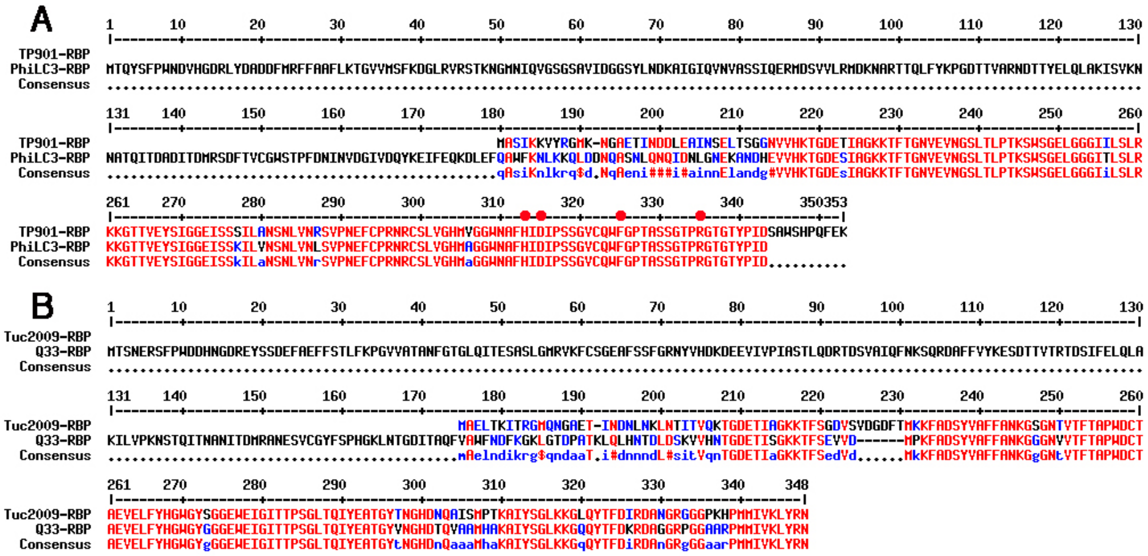

Supplementary Figure S4 Structure and activation of phage p2 baseplate.. **(A)** The interaction between p2 Dit arm & hand domain and the RBP shoulders domain viewed from below the baseplate. **(B)** Structure of the p2 baseplate (Dit, Tal, RBP) viewed from above. **(C)** Lateral view of the p2 baseplate. **(D)** Structure of the p2 baseplate viewed from below. (E) Lateralview of the phage p2 activated baseplate. The RBPs have rotate 220° from the resting state.

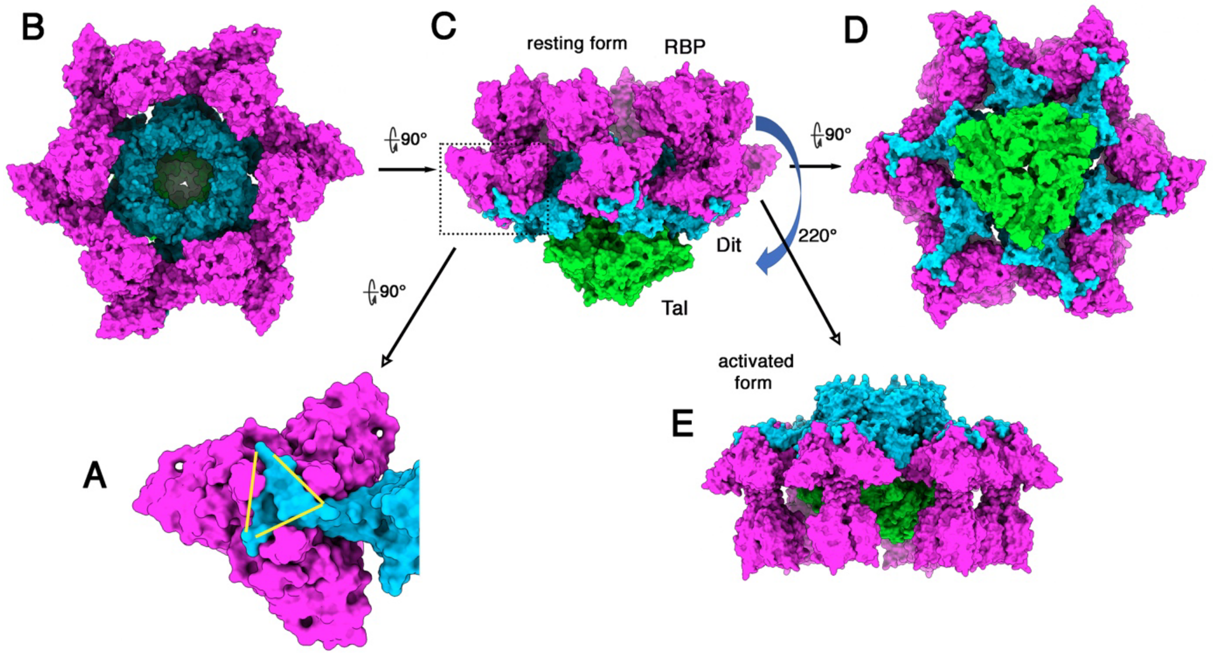

Supplementary Figure S5. Hydrogen bonds established between phage PhiLC3 RBP N-termini and the Dit arm and hand domain.

Hydrogen bonds

	RBP	dist; (Å)	Dit hand
1	B:TYR 17[N]	2.84	E:LEU 160[O]
2	B:ASP 10[N]	2.70	E:PHE 162[O]
3	B:LEU 16[O]	3.36	E:ASN 158[ND2]
4	B:TYR 17[O]	3.66	E:LYS 159[N]
5	B:TYR 17[O]	3.29	E:LEU 160[N]
6	B:ASN 97[OD1]	3.32	E:THR 288[OG1]
1	C:TYR 17[N]	2.83	E:LEU 164[O]
2	C:ASP 10[N]	2.74	E:ILE 166[O]
3	C:TYR 17[O]	2.50	E:LEU 164[N]
4	C:ASP 10[OD2]	2.95	E:SER 165[OG]
1	D:ASP 10[N]	2.69	E:ALA 173[O]
2	D:TYR 17[N]	2.87	E:PHE 170[O]
3	D:ASN 9[OD1]	3.64	E:ALA 173[N]
4	D:ASP 10[O]	2.84	E:ILE 175[N]

Supplementary Figure S6. Buried surface area per residue between PhiLC3 RBP N-termini and the Dit arm and hand domain.

Inaccessible residues
Solvent-accessible residues
HSDC Residues making **H**ydrogen/**D**isulphide bond, **S**alt bridge or **C**ovalent link
Interfacing residues
ASA Accessible Surface Area, Å² **BSA** Buried Surface Area, Å² **ΔG** Solvation energy effect, kcal/mol \|\|\|\| Buried area percentage, one bar per 10%

RBP

Residue	HSDC	ASA	BSA		ΔiG
B:PHE 6		54.57	16.80	||||	0.27
B:PRO 7		86.63	18.48	|||	0.30
B:TRP 8		127.73	8.07	|	-0.03
B:ASN 9		134.70	31.94	|||	-0.14
B:ASP 10	H	70.19	43.47	|||||||	0.03
B:ARG 15		91.47	3.80	|	-0.04
B:LEU 16	H	145.68	79.52	||||||	0.98
B:TYR 17	H	79.47	68.09	|||||||||	0.18
B:ASP 18		86.82	64.89	||||||||	0.41
B:ALA 19		65.57	20.34	||||	0.21
B:ASP 20		79.65	1.72	|	-0.03
B:ASP 21		19.41	1.84	|	-0.02
B:PHE 22		111.27	30.42	|||	0.48
C:PHE 6		50.43	10.90	|||	0.17
C:PRO 7		86.86	13.41	||	0.21
C:TRP 8		132.23	7.89	|	-0.04
C:ASN 9		137.10	44.63	||||	-0.20
C:ASP 10	H	73.83	61.79	|||||||||	-0.18
C:ARG 15		84.96	3.08	|	-0.04
C:LEU 16		151.50	43.66	|||	0.70
C:TYR 17	H	74.31	65.39	|||||||||	0.18
C:ASP 18		83.95	20.35	|||	0.27
C:ALA 19		65.39	22.45	||||	0.24
C:PHE 22		106.82	28.12	|||	0.45
D:PHE 6		41.25	4.34	||	0.07
D:PRO 7		84.51	17.81	|||	0.29
D:TRP 8		125.35	7.74	|	-0.04
D:ASN 9	H	136.85	78.68	||||||	-0.21
D:ASP 10	H	68.16	60.18	|||||||||	-0.19
D:VAL 11		94.11	73.87	||||||||	1.18
D:HIS 12		184.08	77.80	|||||	-0.25
D:ASP 14		100.17	3.79	|	-0.06
D:ARG 15		98.66	5.22	|	-0.06
D:LEU 16		139.46	63.08	|||||	1.01
D:TYR 17	H	70.54	60.08	|||||||||	0.05
D:ASP 18		83.06	35.46	|||||	0.44
D:ALA 19		63.37	17.40	|||	0.18
D:PHE 22		103.67	59.97	||||||	0.96

Dit hand domain

Residue	HSDC	ASA	BSA		ΔiG
E:ASN 148		112.42	13.12	||	-0.15
E:LYS 149		89.05	0.17	|	0.00
E:SER 150		73.22	33.11	|||||	0.25
E:ASN 156		102.09	16.13	||	-0.17
E:ASP 157		81.13	3.79	|	-0.04
E:ASN 158	H	118.79	76.50	||||||	-0.12
E:LYS 159	H	103.80	3.37	|	0.05
E:LEU 160	H	72.67	67.47	||||||||||	0.50
E:LYS 161		146.29	52.58	||||	0.72
E:PHE 162	H	160.99	92.39	|||||	0.82
E:PRO 163		127.83	12.63	|	0.18
E:PHE 172		136.44	54.23	||||	0.80
E:ALA 173		37.39	8.62	|||	0.14
E:PHE 162		160.99	68.60	|||||	1.10
E:PRO 163		127.83	46.93	||||	0.70
E:LEU 164	H	67.21	52.31	||||||||	0.02
E:SER 165	H	70.69	39.34	||||||	0.26
E:ILE 166	H	136.35	82.45	|||||||	0.63
E:LYS 167		134.05	31.91	|||	0.51
E:THR 168		156.10	15.05	|	0.22
E:ILE 166		136.35	53.91	||||	0.86
E:LYS 167		134.05	5.89	|	-0.02
E:THR 168		156.10	44.55	|||	0.11
E:ASP 169		114.37	27.52	|||	0.29
E:PHE 170	H	62.88	46.37	|||||||||	0.08
E:ALA 171		42.29	32.07	|||||||||	0.51
E:PHE 172		136.44	82.22	|||||||	1.25
E:ALA 173	H	37.39	28.78	|||||||||	0.00
E:THR 174		64.10	28.30	|||||	0.38
E:ILE 175	H	80.62	51.25	|||||||	0.54
E:PRO 177		33.03	13.06	||||	0.21

Supplementary Figure S7. Comparison of the structural models of the adhesion devices with nsEM images. The nsEM images are reproduced in part from Figures 1 in references [2] and [3].

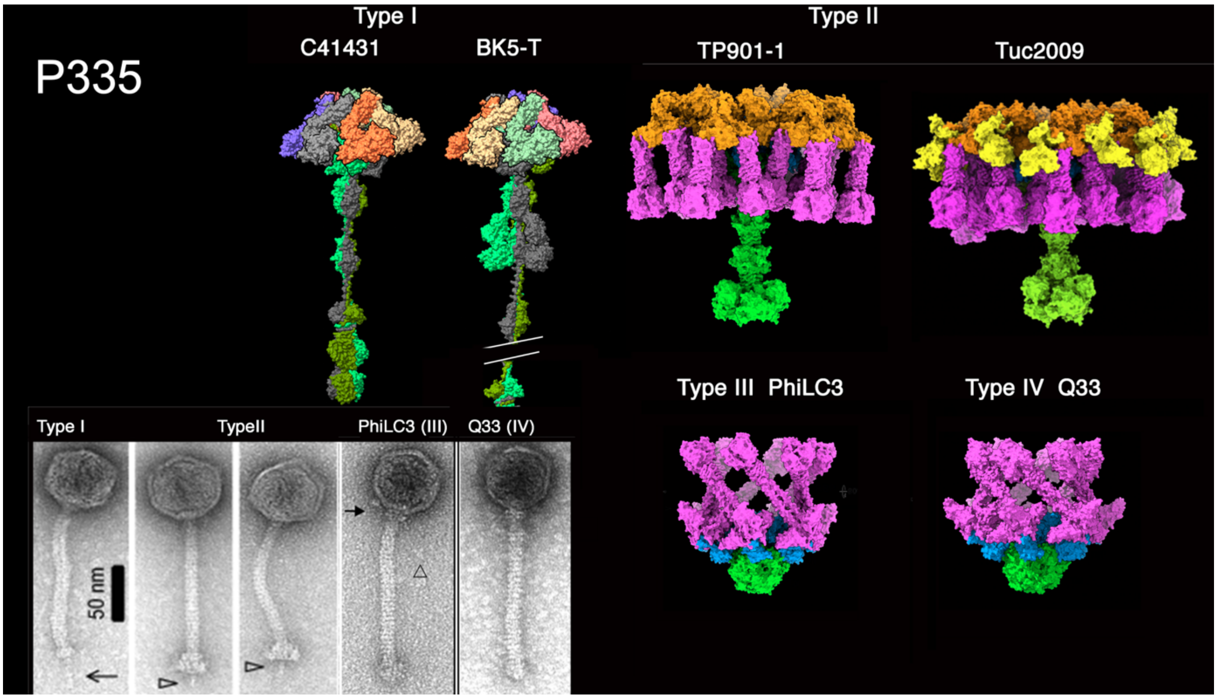

Supplement: Supplementary file 1 [file microorganisms-10-02278-s001.zip › Supplementary-Figures.pdf]
